# Anserine and glucosamine supplementation attenuates the levels of inflammatory markers in rats with rheumatoid arthritis

**DOI:** 10.1186/s13568-020-00987-8

**Published:** 2020-03-20

**Authors:** Jiang Minghao Zhao, Xiang Chen, Ke Cheng, Qingming Shi, Kun Peng

**Affiliations:** Department of Orthopaedics, The Second Affiliated Hospital of Nanchang University, No 1 Minde Road, Donghu District, Nanchang City, 330000 JiangXi Province China

**Keywords:** Rheumatoid arthritis, Anserine, Glucosamine, Antioxidant, Rats

## Abstract

Rheumatoid arthritis (RA) is an autoimmune disorder that affects the joint synovium. Anserine is a functional dipeptide containing methylhistidine and β-alanine, and is present in the brain and skeletal muscle of birds and mammals. Glucosamine is an amino sugar used in the synthesis of glycosylated proteins and lipids. We evaluated the effects of anserine and glucosamine on RA. Rats were assigned into the control group, RA group, anserine group (1 mg/kg), glucosamine group (200 mg/kg), or anserine plus glucosamine group (anserine, 1 mg/kg + glucosamine, 200 mg/kg). Treatment was continued for 45 consecutive days and was administered orally. The serum levels of catalase, glutathione peroxidase (Gpx), superoxide dismutase (SOD), reduced glutathione (GSH), lipid peroxidation, uric acid, nitric oxide, ceruloplasmin, zinc, copper, prostaglandin E_2_ (PGE_2_), matrix metalloproteinase (MMP)-3, tumor necrosis factor (TNF)-α, interleukin (IL)-1β, and IL-6 were assayed. The mRNA and protein levels of nuclear factor (NF)-κB and inducible nitric oxide synthase (iNOS) in synovial tissue were also determined. Anserine plus glucosamine significantly increased the catalase, SOD, Gpx, GSH, and zinc levels compared to the control, anserine, and glucosamine groups. Also, anserine plus glucosamine significantly reduced the PGE_2_, MMP-3, TNF-α, IL-1β, and IL-6 levels compared to the control, anserine, and glucosamine groups. Furthermore, anserine plus glucosamine significantly reduced the mRNA and protein levels of NF-κB and iNOS compared to the control, anserine, and glucosamine groups. Therefore, supplementation of anserine plus glucosamine shows therapeutic potential for RA.

## Introduction

Rheumatoid arthritis (RA) is an autoimmune disorder that affects the joint synovium. Swelling, inflamed joints, stiffness, and pain are the primary symptoms of RA (Choudhary et al. 2018). However, the pathogenetic mechanisms of RA are unclear. Delaying joint function disability is the major therapeutic approach for RA (Wang et al. 2014). Verschueren et al. (2009) have reported the alteration in working situation, normalized physical function and predictors of remission in early rheumatoid arthritis. Researchers have reported the use of biologics in the treatment of rheumatoid arthritis (Curtis and Singh 2011). Liu and Pope (2003) reported that the pannus and hyperplastic synovial tissue erode bone tissue and articular cartilage. Nakamura et al. (2007) showed that tumor necrosis factor (TNF)-α, prostaglandin E_2_ (PGE_2_), nitric oxide (NO), interleukin (IL)-1, and matrix metalloproteinases (MMPs) are important articular components. Therefore, induction of apoptosis and inhibition of cell proliferation are the primary therapeutic approaches for RA.

Anserine is a functional dipeptide containing methylhistidine and β-alanine found in the brain and skeletal muscle of birds and mammals (Kubomura et al. 2010). Tanida et al. (2010) evaluated the impact of anserine on the blood pressure and renal sympathetic nerve activity in rats. Sugiyama et al. (2005) reported that anserine augments the antitumor activity of doxorubicin. Kaneko et al. (2017) showed that anserine improves spatial memory and neurovascular-unit dysfunction in a mouse model of Alzheimer’s disease. Kang et al. (2002) reported that anserine suppresses peroxyl radical-mediated modification of superoxide dismutase (SOD). Glucosamine is an amino sugar used in the synthesis of glycosylated proteins and lipids (Dai et al. 2018), which is produced using wheat, crustacean cytoskeletons, and corn as raw materials (Towheed et al. 2005). Glucosamine supplementation reduced the level of pain of osteoarthritic patients (Reginster et al. 2001). McAlindon et al. (2000) reported that glucosamine and chondroitin synergistically protect against osteoarthritis. Dai et al. (2018) showed that vitamin E and glucosamine exert a synergistic therapeutic effect against RA in a neonatal rat model. Thus, we evaluated the effect of anserine and glucosamine on RA.

## Materials and methods

### Rats and housing

Male albino Wistar strain rats (170–200 g) were purchased from the Animal House of the Department of Orthopaedics, The Second Affiliated Hospital of Nanchang University, No 1 minde road, Donghu district, Nanchang city, JiangXi province, China and maintained in rat cages under a 12/12 h light/dark cycle at 60 ± 5% relative humidity and 25 ± 0.5 °C. All procedures performed in studies involving animals were in accordance with the ethical standards of the Second Affiliated Hospital of Nanchang University at which the studies were conducted. Research proposal was approved on 03/02/2019.

### Model of RA

RA was induced according to Dai et al. (2018; Wang et al. 2015). Briefly, complete Freund’s adjuvant was administered to the rats intradermally. Type II bovine collagen and complete Freund’s adjuvant were mixed at an equal ratio to prepare the adjuvant emulsion. The primary dose of emulsion (1 mg/ml) was administered to the rats intradermally, followed 3 weeks later by incomplete Freund’s adjuvant as a booster.

### Group assignment

Rats were assigned into the normal control group, RA group, anserine (A1131, Sigma-Aldrich, Shanghai, China) group (1 mg/kg), glucosamine (Sigma-Aldrich) group (200 mg/kg), or anserine plus glucosamine (anserine, 1 mg/kg + glucosamine, 200 mg/kg) group. Treatment was continued for 45 consecutive days and was administered orally. Each group contained six rats.

### Biochemical markers

At the end of treatment, rats were sacrificed following anesthetized by using 10% chloral hydrate. The blood was collected by cardiac puncture in a plain bottle, and from which serum was separated for the biochemical analysis. Then, rat was immediately dissected, and synovial tissues were excised and weighed. The serum levels of catalase, glutathione peroxidase (Gpx), SOD, and reduced glutathione (GSH) were determined as described previously (Weydert and Cullen 2010; Baydas et al. 2002). The serum lipid peroxidation level was measured as described elsewhere (Samarghandian et al. 2014). The serum levels of uric acid and nitric oxide (NO) were determined by calorimetric assay (Wu et al. 2018; Van Beezooijen et al. 1998). The serum levels of ceruloplasmin, zinc, copper, PGE_2_, MMP-3, TNF-α, IL-1β, and IL-6 were determined as described previously (Dai et al. 2018).

### Reverse transcriptase-polymerase chain reaction (RT-PCR)

The mRNA and protein levels of nuclear factor (NF)-κB and inducible nitric oxide synthase (iNOS) in synovial tissue were determined according to Dai et al. (2018). Briefly, total RNA was isolated from synovial tissue and transcribed into cDNA, and NF-κB and iNOS mRNA was amplified using specific primers (Table 1).

**Table 1:** List of primers used in real-time polymerase chain reaction (qRT-PCR)

S. no	Gene name	Sense primer	Anti-sense primer
1	iNOS	5′-GTTCTCAAGGCACAGGTCTC-3′	5′-GCAGGTCACTTATGTCACTTATC-3′
2	NF-κB	5′-GAAATTCCTGATCCAGACAAAAAC-3′	5′-ATCACTTCAATGGCCTCTGTGTAG-3′
3	GAPDH	5′-TCCCTCAAGATTGTCAGCAA-3′	5′-AGATCCACAACGGATACATT-3′

### Western blotting

Synovial tissue proteins were resolved by sodium dodecyl sulfate–polyacrylamide gel electrophoresis, transferred onto a membrane, and incubated with antibodies against iNOS (Abcam, ab3523) and NF-κB (Abcam, ab16502) for 12 h. Finally, the blot was incubated with a horseradish peroxidase (HRP)-conjugated secondary antibody (ab6721, Abcam) for 60 min and the protein levels were assayed by enhanced chemiluminescence (ECL) (Zou et al. 2016).

### Immunohistochemistry

At the end of the treatment, rats were anesthetized by 10% chloral hydrate, and sacrificed. The knee synoviums of their hind limbs were harvested and fixed in formalin for 24 h, and embedded in paraffin. Next, the sections were deparaffinized, and rehydrated in xylene and a graded alcohol series. Hydrogen peroxide (3%) was applied to inhibit endogenous peroxidase activity and bovine serum albumin (2%) to block non-specific binding. Synovial tissue was treated with anti-iNOS (Abcam, ab3523) and anti-NF-κB (Abcam, ab16502) antibodies overnight and incubated with an HRP-conjugated antibody for 1 h (Balic et al. 2011). The iNOS and NF-κB expression was analyzed using a confocal microscope (FV300, Olympus, Japan).

### Statistical analysis

Values are mean ± standard deviation and were subjected to analysis of variance (ANOVA) with the Tukey post hoc test. The difference was taken as significant when *P* < 0.05.

## Results

### Biochemical marker levels in RA rats

**Fig. 1:**
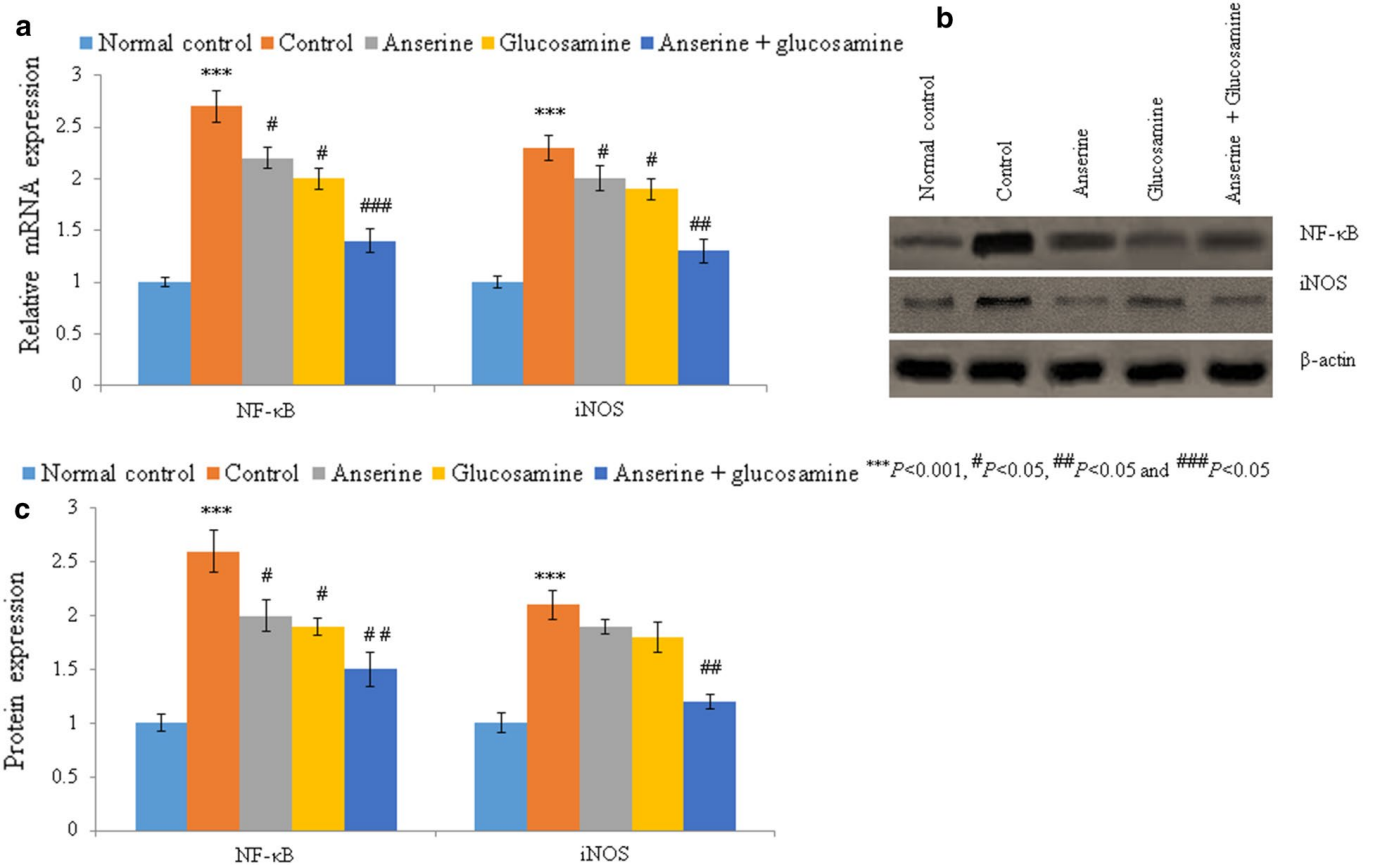
Anserine plus glucosamine supplementation reduced the mRNA and protein levels of NF-κB and iNOS in synovial tissue. **a** mRNA levels of NF-κB and iNOS. **b** Western blots of NF-κB and iNOS. **c** Densitometric analysis of the data in **b**

**Fig. 2:**
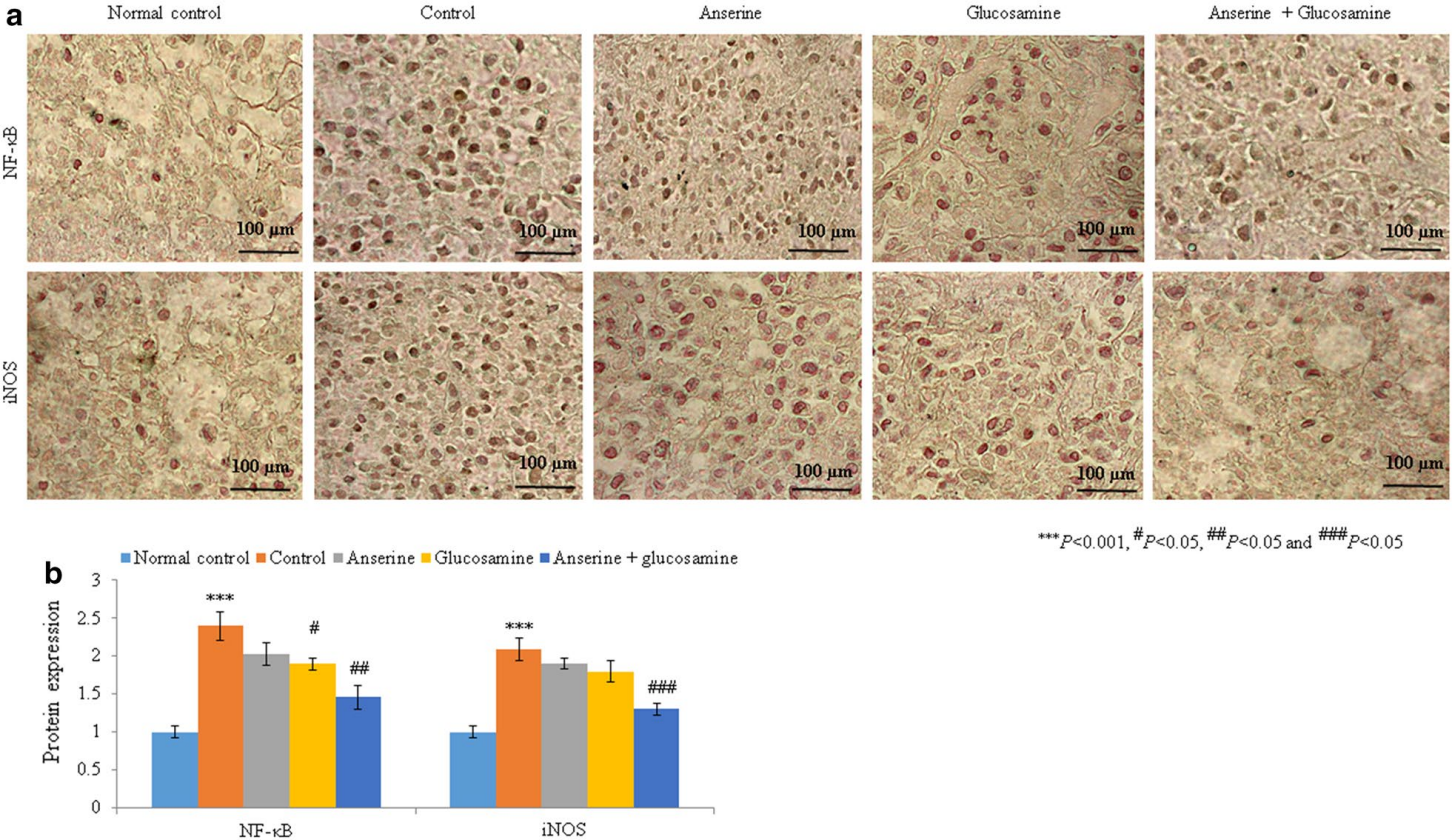
Anserine plus glucosamine supplementation reduced the protein levels of NF-κB and iNOS in synovial tissue. **a** Immunohistochemical analysis of NF-κB and iNOS. **b** Analysis of the protein levels in **a**. Scale bar is 100 µm

We evaluated the effect of anserine and glucosamine on RA. The SOD, catalase, Gpx, and GSH levels were reduced by 63.6%, 74.2%, 73.3%, and 63.4%, respectively, in the RA group compared to the normal control group (Table 2, *P* < 0.05). Lipid peroxidation, NO, uric acid, ceruloplasmin, and copper were increased by 431.3# 288%, 200%, 126.7%, and 92.3%, respectively (Table 2, *P* < 0.05), while the zinc level was decreased by 158.3% (Table 2, *P* < 0.05). The MMP-3, PGE_2_, TNF-α, IL-1β, and IL-6 levels were increased by 328.6%, 251.3%, 68.9%, 126.7%, and 166.7%, respectively (Table 2, *P* < 0.05). The mRNA and protein levels of NF-κB and iNOS were also significantly increased (Figs. 1 and 2, *P* < 0.05).

**Table 2:** Effect of anserine and glucosamine on the biochemical markers in rheumatoid arthritis induced rat model

Markers	Normal control	Control	Anserine (1 mg/kg)	Glucosamine (200 mg/kg)	Anserine (1 mg/kg) + Glucosamine (200 mg/kg)
SOD (U/ml)	365.2 ± 21.4	133.1 ± 5.2^***^	197.4 ± 7.5^#^	213.2 ± 11^#^	313.4 ± 16^###^
Catalase (U/ml)	13.2 ± 0.8	3.4 ± 0.18^***^	5.9 ± 0.2^#^	6.2 ± 0.3^#^	10.6 ± 0.5^###^
Gpx (U/ml)	0.45 ± 0.01	0.12 ± 0.005^***^	0.21 ± 0.02^#^	0.25 ± 0.01^#^	0.38 ± 0.01^###^
GSH (nmol/ml)	0.41 ± 0.03	0.15 ± 0.01^***^	0.22 ± 0.01^#^	0.24 ± 0.01^#^	0.36 ± 0.02^###^
MDA (nmol/ml)	0.32 ± 0.01	1.7 ± 0.1^***^	1.1 ± 0.06^#^	0.9 ± 0.05^##^	0.51 ± 0.03^###^
NO (ng/ml)	0.25 ± 0.02	0.97 ± 0.05^***^	0.73 ± 0.05^#^	0.62 ± 0.07^#^	0.44 ± 0.08^###^
Uric acid (mg/ml)	0.23 ± 0.01	0.69 ± 0.03^***^	0.52 ± 0.01^#^	0.54 ± 0.04^#^	0.35 ± 0.03^###^
Ceruloplasmin (mg/ml)	0.15 ± 0.01	0.34 ± 0.01^***^	0.26 ± 0.01^#^	0.31 ± 0.01	0.17 ± 0.04^##^
Copper (µg/ml)	0.13 ± 0.01	0.25 ± 0.01^**^	0.23 ± 0.01	0.21 ± 0.01	0.17 ± 0.03^#^
Zinc (µg/ml)	0.31 ± 0.02	0.12 ± 0.01^***^	0.15 ± 0.01	0.14 ± 0.01	0.24 ± 0.02^##^
MMP-3 (ng/ml)	52.5 ± 3.2	225 ± 15^***^	171.3 ± 7.1^#^	156 ± 8^#^	81 ± 6.4^###^
PGE_2_ (pg/ml)	27.1 ± 1.2	95.2 ± 5^***^	72.2 ± 3.4^#^	71.4 ± 4.5^#^	38.3 ± 2^###^
TNF-α (U/ml)	2.9 ± 0.12	4.9 ± 0.2^***^	4.1 ± 0.1^#^	3.9 ± 0.13^#^	3.2 ± 0.2^##^
IL-1β (U/ml)	1.5 ± 0.01	3.4 ± 0.2^***^	2.7 ± 0.05^#^	2.5 ± 0.12^#^	1.8 ± 0.05^###^
IL-6 (U/ml)	2.7 ± 0.12	7.2 ± 0.21^***^	6.1 ± 0.2^#^	5.1 ± 0.15^#^	3.3 ± 0.12^##^

^***^P < 0.001, ^#^*P *< 0.05, ^##^*P *< 0.05 and ^###^*P *< 0.05

### Effect of anserine on biochemical and molecular markers in RA rats

The SOD, catalase, Gpx, and GSH levels were increased by 48.3%, 73.5%, 75%, and 46.7%, respectively, in the anserine group compared to the control group (Table 2, *P* < 0.05). Lipid peroxidation, NO, uric acid, ceruloplasmin, and copper were reduced by 35.3%, 24.7%, 24.6%, 23.5%, and 8%, respectively, while the zinc level was increased by 25% (Table 2, *P* < 0.05). The MMP-3, PGE_2_, TNF-α, IL-1β, and IL-6 levels were reduced by 23.8%, 24.1%, 16.3%, 20.6%, and 15.3%, respectively (Table 2, *P* < 0.05). The mRNA levels of NF-κB and iNOS were reduced by 18.5% and 13% respectively, whereas protein levels of NF-κB and iNOS were reduced by > 9% compared to the control group (Figs. 1 and 2, *P* < 0.05).

### Effect of glucosamine on biochemical and molecular markers in RA rats

The SOD, catalase, Gpx, and GSH levels were increased by 60.2%, 82.4%, 108.3%, and 60%, respectively, in the glucosamine group compared to the control group (Table 2, *P* < 0.05). Lipid peroxidation, NO, uric acid, ceruloplasmin, and copper were reduced by 47%, 36.1%, 21.7%, 8.8%, and 20%, respectively, while the zinc level was increased by 16.7% (Table 2, *P* < 0.05). The MMP-3, PGE_2_, TNF-α, IL-1β, and IL-6 levels were reduced by 30.7%, 25%, 20.4%, 26.5%, and 29.2%, respectively (Table 2, *P* < 0.05). The mRNA levels of NF-κB and iNOS were reduced by 25.9% and 17.4% respectively, whereas protein levels of NF-κB and iNOS were reduced by > 13% in the glucosamine group compared to the control group (Figs. 1 and 2, *P* < 0.05).

### Synergistic effect of anserine plus glucosamine on biochemical and molecular markers in RA rats

The SOD, catalase, Gpx, and GSH levels were increased by 135.5%, 211.8%, 216.7%, and 140%, respectively, in the anserine plus glucosamine group compared to the control group (Table 2, *P* < 0.05). Lipid peroxidation, NO, uric acid, ceruloplasmin, and copper were reduced by 70%, 54.6%, 49.3%, 50%, and 32%, respectively, while the zinc level was increased by 100% (Table 2, *P* < 0.05). The MMP-3, PGE_2_, TNF-α, IL-1β, and IL-6 levels were significantly reduced in the anserine plus glucosamine group (Table 2, *P* < 0.05). The mRNA and protein levels of NF-κB and iNOS were also significantly reduced in the anserine plus glucosamine group compared to the control, anserine, and glucosamine groups (Figs. 1 and 2, *P* < 0.05).

## Discussion

We evaluated the effect of anserine and glucosamine on RA. A low level of cellular antioxidants and increased production of free radicals are implicated in RA (van Vugt et al. 2008). A higher rate of membrane fatty acid oxidation results in elevated levels of lipid peroxyl radicals. Ozturk et al. (1999) reported that the rate of lipid peroxidation is higher in patients with RA compared to healthy persons. Anserine and glucosamine have free radical-scavenging activity and inhibit lipid peroxidation (Wu et al. 2003; Tiku et al. 2007). Supplementation of glucosamine with vitamin E reduced lipid peroxidation in neonatal rats (Dai et al. 2018). In this study, anserine plus glucosamine decreased the level of lipid peroxidation compared to anserine, glucosamine, and the control.

Oxidative injury and inflammation increase the level of prostaglandin (Bae et al. 2003). MMP-3 produced by synovium-lining cells activates pro-collagenases, leading to destruction of cartilage proteoglycans and type IX collagen (Miller et al. 2009). The levels of PGE_2_ and MMP-3 in patients with RA are reduced by treatment with glucosamine (Nakamura et al. 2007). A high MMP-3 level is indicative of radiological damage and cartilage degradation (Ally et al. 2013). Supplementation of glucosamine with vitamin E reduced PGE_2_ and MMP-3 levels in neonatal rats (Dai et al. 2018). In this study, anserine plus glucosamine decreased the level of MMP-3 compared to anserine, glucosamine, and the control. Indeed, the MMP-3 level is reportedly reduced by glucosamine supplementation (Nakamura et al. 2002).

Antioxidants are closely correlated with the levels of oxidants and lipid peroxidation (Gupta et al. 2009). Reduced levels of catalase, SOD, Gpx, and GSH were noted in the control (RA) rats but were restored by anserine plus glucosamine to almost normal ranges. Dai et al. (2018) have reported the glucosamine with vitamin E supplementation restored the levels of catalase, SOD, Gpx, and GSH in neonatal rats. Sugiyama et al. (2005) reported that anserine augments the antitumor activity of doxorubicin. Kang et al. (2002) showed that anserine suppresses peroxyl radical-mediated SOD modification. Dai et al. (2018) reported that vitamin E and glucosamine exert a synergistic therapeutic effect against RA in neonatal rats. Ali et al. (2019) have reported the niclosamide supplementation exert anti-rheumatoid activity in collagen-induced arthritis in rat model. Higher levels of copper and ceruloplasmin (Amancio et al. 2003) and uric acid (Choe and Kim 2015) and a lower level of zinc were noted in rats with RA (Mierzecki et al. 2011), possibly due to an elevated level of IL-1 (Nemeth et al. 2002). Wang et al. (2019) have reported that the curcumin attenuates collagen-induced rat arthritis through apoptotic and anti-inflammatory effects. Researchers have reported that the evodiamine reduces adjuvant-induced arthritis in rats through the inhibition of synovial inflammation and restoring the Th17/Treg balance (Zhang et al. 2020). Supplementation of telmisartan with etanercept attenuates anemia associated with rheumatoid arthritis in rats (Hasanin and Mohamed 2020).

In this study, anserine plus glucosamine restored the levels of copper, ceruloplasmin, and zinc to almost the normal ranges. Taken together, our findings suggest that anserine plus glucosamine supplementation shows therapeutic potential for RA.

## Data Availability

Corresponding author could provide the all experimental data on valid request.
